# MEDEP: Maintenance Event Detection for Multivariate Time Series Based on the PELT Approach

**DOI:** 10.3390/s22082837

**Published:** 2022-04-07

**Authors:** Milot Gashi, Heimo Gursch, Hannes Hinterbichler, Stefan Pichler, Stefanie Lindstaedt, Stefan Thalmann

**Affiliations:** 1 Pro2Future GmbH, 4040 Linz, Austria; 2 Know-Center GmbH, 8010 Graz, Austria; hgursch@know-center.at (H.G.); lindstaedt@tugraz.at (S.L.); 3 Fronius International, 4643 Pettenbach, Austria; hinterbichler.hannes@fronius.com (H.H.); pichler.stefan@fronius.com (S.P.); 4 Business Analytics and Data Science Center, University of Graz, 8010 Graz, Austria

**Keywords:** event detection, welding industry, predictive maintenance, maintenance event detection, change point detection

## Abstract

Predictive Maintenance (PdM) is one of the most important applications of advanced data science in Industry 4.0, aiming to facilitate manufacturing processes. To build PdM models, sufficient data, such as condition monitoring and maintenance data of the industrial application, are required. However, collecting maintenance data is complex and challenging as it requires human involvement and expertise. Due to time constraints, motivating workers to provide comprehensive labeled data is very challenging, and thus maintenance data are mostly incomplete or even completely missing. In addition to these aspects, a lot of condition monitoring data-sets exist, but only very few labeled small maintenance data-sets can be found. Hence, our proposed solution can provide additional labels and offer new research possibilities for these data-sets. To address this challenge, we introduce MEDEP, a novel maintenance event detection framework based on the Pruned Exact Linear Time (PELT) approach, promising a low false-positive (FP) rate and high accuracy results in general. MEDEP could help to automatically detect performed maintenance events from the deviations in the condition monitoring data. A heuristic method is proposed as an extension to the PELT approach consisting of the following two steps: (1) mean threshold for multivariate time series and (2) distribution threshold analysis based on the complexity-invariant metric. We validate and compare MEDEP on the Microsoft Azure Predictive Maintenance data-set and data from a real-world use case in the welding industry. The experimental outcomes of the proposed approach resulted in a superior performance with an FP rate of around 10% on average and high sensitivity and accuracy results.

## 1. Introduction

Predictive Maintenance (PdM) is one of the most prominent industrial applications of data-driven technologies and key to the smart manufacturing concepts, promising many benefits such as optimized maintenance scheduling, resource optimization, and improved decision support [1]. PdM models are typically used to predict future failures due to the wearing out of components and thus provide the opportunity to perform maintenance proactively. The main reasons for the interest of researchers and industry alike in PdM in recent years are the relevance and influence of maintenance on production cost and quality [2], the increased information base due to the availability of cheap and powerful sensor technology [3], and huge advances in artificial intelligence (AI) [4]. In general, maintenance costs are an aspect that make up the majority of operating costs and can vary between 15% and 60% depending on the type of industry [5]. Consequently, PdM helps to reduce maintenance costs without increasing the risk of downtimes. For instance, Han et al. [6] introduced a Remaining Useful Life (RUL)-driven PdM approach that reduced the maintenance costs by 4% compared to scheduled maintenance.

Building PdM models requires a comprehensive amount of condition monitoring data describing the operation conditions of the machinery and maintenance data documenting maintenance events. Usually, the condition monitoring data are collected by sensors embedded in smart manufacturing systems. Yet, the collected maintenance data mainly consist of feedback from shop-floor workers, and motivating the shop-floor workers to provide this feedback is a big challenge. This is often neglected due to time constraints. Consequently, maintenance data are often incomplete or even completely missing [7,8]. One way to tackle the challenge of missing or incomplete maintenance documentation is to automatically detect the maintenance events based on their manifestation in monitoring data like time series collected from sensors in the machines. Anomaly detection approaches are suitable for these event detection tasks and can be found in many topics within manufacturing including defect detection [9,10], fault detection [11,12], or maintenance event detection [7]. This research aims to automatically detect performed maintenance actions in monitoring data to create comprehensive data-sets. Subsequently, the completed maintenance and condition monitoring data can be used to build suitable PdM models, which in turn will help to facilitate maintenance scheduling, optimize manufacturing processes, and enhance product quality. We will show that Change-point detection (CPD) is a promising technique for maintenance event detection.

Researchers investigated the application of CPD techniques as event detection approaches. CPD is a common and promising approach to tackle this challenge as they aim to detect abrupt changes in time series [13]. The Pruned Exact Linear Time (PELT) in particular is a state-of-the-art offline CPD method that provides accurate event detection outcomes as a result of its binary segmentation and the lower computational complexity it offers compared to exact search methods [14]. The main advantage of PELT is its use of pruning to reduce computational costs without affecting the accuracy of the segmentation results. However, a drawback of event detection approaches in general, and CPD in particular, is their tendency to predict a large number of False-Positive (FP) events [15,16,17]. FP events add additional noise in case the list of events is used as an input for other algorithms. Moreover, a large number of FP events can hinder the application of such models in real scenarios, thus decreasing the usefulness of these approaches [17]. To address this challenge, we propose MEDEP as a novel framework based on PELT for multivariate time series. The experimental results are evaluated using two different manufacturing use cases. As a result, MEDEP promises high accuracy event detection results at a low FP rate. The provided low FP rate is a crucial aspect when aiming to integrate these approaches in real-world use cases, and promises increased potential for higher acceptance and trust in these approaches, and in turn a high application rate. We consider this an important contribution to the field of detecting maintenance interventions in manufacturing. Note that the focus of this work is only on detecting maintenance intervention from sensor data to create comprehensive data-sets, thus providing a foundation for PdM research in the future; however, the PdM is beyond the scope of this paper.

The main contributions of this work are summarized by the following two aspects:

1. The design of MEDEP as a novel framework based on PELT to detect maintenance events within sensor data represented as multivariate time series. The PELT approach is extended with a post-filtering heuristic method that consists of two consecutive steps of mean ratio and distribution threshold filtering that validate suspected maintenance events, ensuring a high accuracy rate at a very low FP rate.
2. A novel complexity-estimate-based metric [18] for time series is proposed to extract relevant knowledge concerning maintenance event interventions. The metric helps to select the most informative sensors concerning the performed maintenance actions by searching for the sensor with the largest difference of the complexity estimate before and after the performed maintenance action. This is based on the hypothesis that the sensor data before performing the maintenance action will have more and larger peaks and valleys due to the worn-out component. This metric is used for feature selection for PELT and to select the appropriate feature for the distribution threshold analysis within the post-filtering method.

## 2. Theoretical Background

In the age of smart industrial diagnostics, multiple sensors are embedded within machines to collect condition monitoring data [9]. This provides the foundation to develop data-driven models to understand, support, and automate manufacturing processes. Despite the huge advances of PdM, many companies struggle to build suitable PdM models. The major reason for this struggle and also the major barrier of introducing PdM in the industry is the lack of suitable data. In particular, the process of collecting maintenance data is challenging as it requires human feedback to document performed maintenance activities. Usually, the shop-floor workers focus on the execution of maintenance actions so that manufacturing continues as soon as possible, and the documentation of their works is only a secondary concern. In many cases, the documentation of maintenance actions is performed retrospectively; thus, a lot of details are not included.

Sensor data are typically more complete than the feedback provided by humans. Based on this observation, several researchers proposed technical approaches for event detection to overcome the missing human feedback as a major barrier of introducing PdM. The collected sensor data can serve the purpose of anomaly detection in general, defect detection [9,10,19], failure detection [11,12], or maintenance events detection [7] in particular. Event detection for specific components in large machines is challenging due to the high degree of complexity inherent to the large number of components and environmental factors influencing the health state of the machines and their components [1]. Data-driven models are seen as a promising solution to tackle these challenges. Supervised, semi-supervised, and unsupervised machine learning methods have found their applications for anomaly detection in manufacturing.

Supervised approaches are widely applied and usually provide good results. Typical application examples of supervised learning in manufacturing can be found in [9,20,21]. These approaches require large labeled data-sets for their training where the condition monitoring data are annotated with known maintenance events indicating the true health conditions of the machine. However, such annotations are often incomplete and not available in real-world use cases [22,23]. Semi-supervised approaches are a promising way to overcome the challenge of incompletely annotated data-sets [24]. The main characteristic of semi-supervised approaches is the repeated training with a labeled subset of all maintenance events to continuously improve detection or predictive results. For semi-supervised modeling, at least a partly annotated training data-set representing the healthy state of machines and components has to be available. However, this is hard to assure as machines continuously degrade or even crash, and such crashes might affect the condition monitoring system and, therefore, the collected data [25].

Unsupervised approaches can overcome these issues since they learn solely from condition monitoring data and neither require labeled nor only healthy system data [26,27,28]. The focus in this research field is targeted towards identifying abnormal patterns that can be exploited for fault detection or event detection knowledge. However, their application in maintenance event detection is less explored. The knowledge acquired in fault and defect event detection models is mostly used as input for maintenance decision making. Nevertheless, anomaly detection for maintenance event detection has been receiving more attention recently [7,8,29]. In this context, research mostly focuses on the detection of abnormal patterns using sensor data complemented by a human-in-the-loop setup to validate the detection results. For instance, Moens et al. [7] introduce an interactive dashboard for event detection in sensor data. This approach is based on a matrix profile as its motif discovery technique and requires human feedback or intervention to label correct maintenance events. This work showed that maintenance events could be detected and correctly labeled with limited feedback from the human expert. De Benedetti et al. [12] proposed an anomaly detection approach detecting anomalies in photovoltaic systems based on artificial neural networks to generate predictive maintenance alerts. Furthermore, Theodoropoulos et al. [30] evaluated Deep Learning-based approaches in a maritime industry sustainability. The work [30] showed that 1D-CNN models can successfully deduce important properties, i.e., component decay and status, in different time horizons. In contrast to benchmark ML approaches, the proposed methodology showed efficiency in the detection of defect patterns for small degradations. Susto et al. [31] compared state-of-the-art anomaly detection approaches using different industrial use cases. As a result, Local Outlier Factor (LOF) [32] outperforms other approaches in terms of outlier and event detections.

However, these approaches are not straightforward and require human feedback [7] to validate the detected events that lead to extra effort from shop-floor workers. A common challenge of anomaly detection techniques is the tendency to detect a large number of false positives (FPs) [15,16,17]. The tuning of hyper-parameters in these models helps to reduce the FP rate, but usually at the expense of the sensitivity rate, reducing the performance in the detection of real events [15,16]. The detection of maintenance actions requires high accuracy results and, therefore, also low FP rate. High FP rates hinder the application of such models in real-world use cases, thus decreasing the usefulness of event detection approaches [17]. Therefore, MEDEP, presented in this work, is designed as a novel event detection approach that tackles all the aforementioned challenges in the context of maintenance event detection.

We use PELT [14] as the CPD component in MEDEP to achieve high detection accuracy results, especially in terms of the sensitivity rate, i.e., the detection of true events. PELT is an offline CPD approach achieving accurate results due to binary segmentation while at the same time posing less computational cost as exact search methods. The main advantage of PELT is that it uses pruning to reduce the computational costs while not affecting the accuracy of the segmentation results [14]. We propose MEDEP as a fully automated framework that detects maintenance events with minimal human input. MEDEP takes advantage of unsupervised learning techniques, in particular CPD, which is at its core. Furthermore, MEDEP extracts additional knowledge from a subset of labeled data used mainly for the initial training of hyper-parameters. A key feature of MEDEP is that it needs only a very small set of labeled training data, thus minimizing the need for manually labeled training data. Finally, MEDEP tackles the challenge of a high FP rate with a post-filtering approach. This approach is introduced in Section 4.3.

## 3. Problem Definition and Data Description

MEDEP is evaluated on two different data-sets; the first one is a comprehensive public data-set published by Microsoft [33] and called Microsoft Azure Predictive Maintenance. It is designed for PdM application and was collected in the semiconductor industry [34]. The second data-set was collected by a major Austrian welding equipment manufacturer. The Microsoft Azure Predictive Maintenance will be referred to hereafter as Use Case 1, and the work on the data-set collected by the welding manufacturer is referred to as Use Case 2.

### 3.1. Use Case 1—Microsoft Azure Predictive Maintenance Data-Set

Use Case 1 benefits from the comprehensiveness of the Microsoft Azure Predictive Maintenance data-set, since this data-set is complete in terms of sensor data, error log, and maintenance data. Therefore, the evaluation numbers achieved in Use Case 1 are thought to be of high quality since there are no unlabeled maintenance events expected in the data-set. The data-set is suitable for event detection and consists of machine conditions and usage data formed from telemetry records, error records, and maintenance logs representing the failure data. Especially, the completeness of the maintenance logs elevates this data-set over the data-sets collected in other applications where the maintenance logs are potentially incomplete.

Machine conditions and usage time series data consist of hourly averages of voltage, rotation, pressure, and vibration collected from 100 machines in the year 2015. In total, the data-set contains 8761 records. Each record consists of the aforementioned four values, a timestamp, and a machine identifier, as can be seen in Table 1. The failure history of four components named comp1, comp2, comp3, and comp4 contains 761 records describing around eight failures per machine in the year 2015. Failures lead to crashes or machine shut down, thus forcing the replacement of the failed components. Each failure record contains information about the failed component, the timestamp, and the affected machine. Furthermore, the error log contains a list of 3919 errors encountered by the machines while in operating conditions. An error’s presence does not cause a crash or force the machine to shut down; therefore, errors are not considered as a failure. Moreover, the timestamps of errors are rounded to the nearest hour to fit the machine conditions and usage data collected hourly. There are five types of errors numbered from “error1” to “error5”. Each recorded error consists of the encountered error type, machine, and timestamp. Further information regarding the components and errors was removed during the anonymization by the data-set’s publisher and is therefore not available.

### 3.2. Use Case 2-Welding Industry

Use Case 2 considers an industrial welding application in integrated manufacturing lines. The objective of Use Case 2 is to detect maintenance events from sensor data. There are components of a modern industrial welding machine that are not replaced based on a preventive scheme due to the high cost, complex replacement, and rare failure, but are replaced only when problems occur. Nevertheless, many of these components are subject to wear, and their replacement is a complex maintenance activity conducted by trained shop-floor workers in a couple of minutes. This forces machine downtime, thus strongly affecting the performance of the manufacturing process. A prediction of when the components need to be replaced would be highly desirable to schedule component replacements in advance. Since welding process data and machine condition data are automatically collected and provided, the objective of Use Case 2 is to detect conducted component maintenance actions from such data.

The data in Use Case 2 were collected in a welding process involving eight different machines in the time from June 2020 to June 2021. However, for the evaluation of the MEDEP, only one machine was used due to it offering the highest data quality. Statistical features including the mean, variance, standard deviation (std), kurtosis, and skewness of sensor data are estimated based on the welded parts over time. Furthermore, errors are counted over the welded parts. Additionally, the duration of welded parts and component logs are used as features for the model. However, due to the tight organization of the manufacturing process, only a subset of the maintenance actions are documented directly after a maintenance action is conducted. Domain experts investigated the collected welding and condition monitoring data to retrospectively complete the list of maintenance actions. As a result, nine maintenance events were described within the machine that is used to evaluate the MEDEP. This retrospectively defined list is used in this evaluation as ground truth.

## 4. Maintenance Event Detection Framework

**Figure 1:**
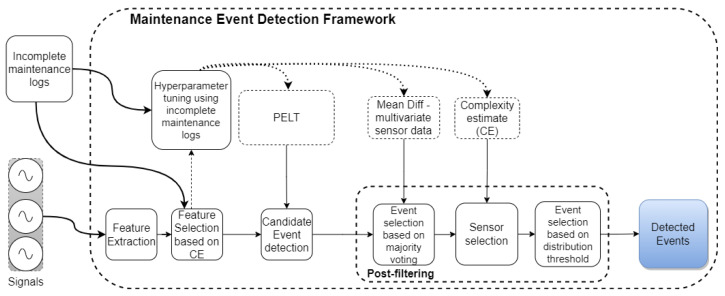
Maintenance event detection framework. Consisting of the following steps: Feature extraction and feature selection based on complexity estimate (CE), hyper-parameter tuning using partly maintenance logs, initial event detection based on the PELT, and post-filtering (mean and distribution analysis) to reduce FP rate.

Figure 1 shows the proposed MEDEP setup consisting of feature extraction and selection, hyper-parameter tuning to identify the global optimum, candidate event detection based on the PELT, and candidate events validation based on a post-filtering heuristic approach. The post-filtering approach consists of two consecutive steps of candidate events validation based on mean ratio and candidate event validation based on distribution analysis. The post-filtering is responsible for removing FP events, thus assuring high accuracy and high sensitivity, resulting in a high detection performance of the real maintenance events. Based on background knowledge and the assumption that the wear of different components shows different deviations in sensor data, the framework is trained and evaluated for each component separately.

### 4.1. Feature Extraction and Selection

The pre-processing is split into feature engineering and feature selection. The feature engineering defines features based on the available input data so that these features can be inputed in PELT. This starts with aggregating sensor data to exclude noise and highlight only relevant changes and was conducted for every use case separately. In Use Case 1, the feature engineering is based on the previous works by Microsoft [33], Cardoso and Ferreira [35]. The telemetry data of voltage, vibration, rotation, and pressure is aggregated into 3-h blocks to catch short-term knowledge and in 24 h blocks to catch long-term knowledge. Any errors occurring during the 3 h blocks are counted, and the error count is used as a feature. In Use Case 2, the sensor data are aggregated per welded part. This was achieved by calculating the statistical features mean, standard deviation, variance, kurtosis, and skewness of the sensor data collected during the welding of a single part. Domain experts have suggested these features. The number of errors during the time it takes to weld the part was also used as a feature, analogous to Use Case 1. The features are normalized by subtracting the mean and scaling to unit variance in both use cases to assure similar characteristics for similar events and to exclude the magnitude effect.

The feature selection is based on the complexity estimate (CE) of the complexity-invariant distance for time series as defined by Batista et al. [18]. The complexity estimate CE(X) of a univariate time series $X=[x_{1},x_{2},\ldots ,x_{N}]$ with a length of *N* is defined by Batista et al. [18] (Equation (1)) as

(1) $$CE\left(X\right)=\sqrt{\sum_{i=1}^{N-1}{\left(x_{i}-x_{i+1}\right)}^{2}}.$$

For the feature selection, the CE(X) is evaluated before and after a known maintenance event for all input feature candidates. The ratio

(2) $$ce\_ratio=\frac{CE(X_{before})}{CE(X_{after})}$$

is calculated for each known maintenance event. Then, features with a high ce_ratio are selected. The best subset of features is selected based on the backward elimination where the features that did not show significant drops in ce_ratio are eliminated. The selected features in Use Case 1 and Use Case 2 are shown in Table 2.

### 4.2. Hyper-Parameter Tuning

The data-set is split into train and test data. In Use Case 1, this split is conducted using a machine-based approach, meaning that the data of 80% of the machines are selected for training, and data from the other 20% of machines are used as test data. The assignment of machines to either the training or the test data-set is carried out randomly. This split on a per-machine basis is carried out to evaluate the transferability of the model over different machines. However, in Use Case 2, only one machine is used to evaluate the MEDEP; therefore, a time-based split is conducted, where 60% are train and 40% test data. GridSearchCV is used to search for the optimum of all hyper-parameters, namely penalty and cost function as a parameter of PELT approach, window size, mean ratio, and distribution threshold required for post-filtering analysis. The complete list of tuned hyper-parameters concerning Use Cases 1 and 2 is shown in Table 3. The selection of the best hyper-parameters is conducted based on the highest sensitivity and lowest FP rate, where the sensitivity is prioritized. In other words, this metric aims to find the optimal hyper-parameters that deliver the highest sensitivity first, and in case that multiple parameter sets deliver similar sensitivity results, the lowest FP rate is used to select the optimal hyper-parameter set.

### 4.3. Maintenance Event Detection

The initial event detection is conducted with PELT due to its high accuracy and low computational costs. The L2 regularization cost function is used, and the penalty parameter is optimized separately for each component in both use cases, as described in Section 4.2. While a high sensitivity was achieved, still an also high FP rate persisted. Therefore, the post-filtering steps are designed to mitigate this issue.

**Figure 2:**
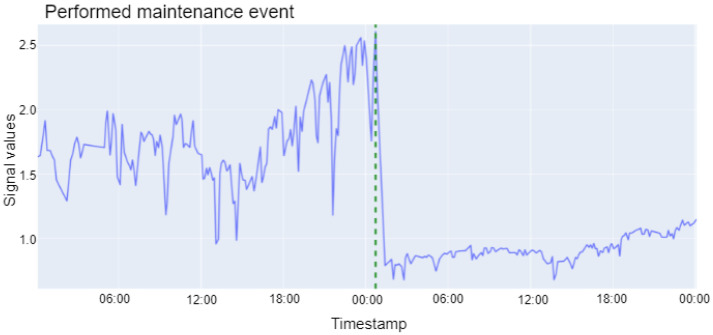
An example of performed maintenance event. The maintenance intervention is indicated by the dashed vertical line in green. The sensor yields higher absolute values and higher variability before the maintenance compared to the sensor values after the maintenance event is performed.

The post-filtering is carried out to reduce the number of FPs in the result of PELT. The post-filtering methods are motivated by the fact that the most informative sensors concerning maintenance events show larger variability and higher absolute values before the performed maintenance event due to worn-out components. This can be seen in the more significant peaks and valleys before the maintenance event, as depicted in Figure 2. Note that the example shown in Figure 2 is a maintenance intervention that can be demonstrated visually and could help to understand the foundation of the proposed approach; however, not every intervention can be shown visually in such an exemplary way. The sensor in Figure 2 shows lower variability, and the expected sensors values also drop on average after the component is replaced. Therefore, the ce_ratio as defined in Equation (2) is employed to capture variability changes and the ratio of the sensor’s mean value before and after a potential maintenance event. The acceptance thresholds of ce_ratio and the mean ratio for real maintenance events are tuned using known maintenance events in the training data and are an integral part of the post-filtering.

The post-filtering includes two consecutive steps. The first step is the calculation of the ratio of the sensor’s mean value before and after a potential maintenance event for all features. A majority vote on the calculated mean ratios decides if the potential maintenance event is filtered out or not. The second step is centered around a distribution threshold applied to the most informative feature to reduce the FP rate further. This begins by selecting the most informative feature based on the sum of ce_ratio and ci_ratio as given in Equations (2) and (3), respectively. CI in Equation (3) is the confidence interval range of CE(X). ci_ratio is calculated as a ratio of CI before and after the performed maintenance events. Once the most informative feature is selected, the threshold separating the distribution of the real maintenance events and the distribution of the FP maintenance events is defined. The threshold is defined using the kernel density estimate [36] and the root finding algorithm [37]. The central root between the means of two distributions is selected as the intersection point and consequently as the deciding threshold. These parameters are entirely tuned using only training data. Once it is trained, the post-filtering is applied to every potential maintenance event detected by PELT.

(3) $$ci\_ratio=\frac{CI(X_{before})}{CI(X_{after})}$$

**Figure 3:**
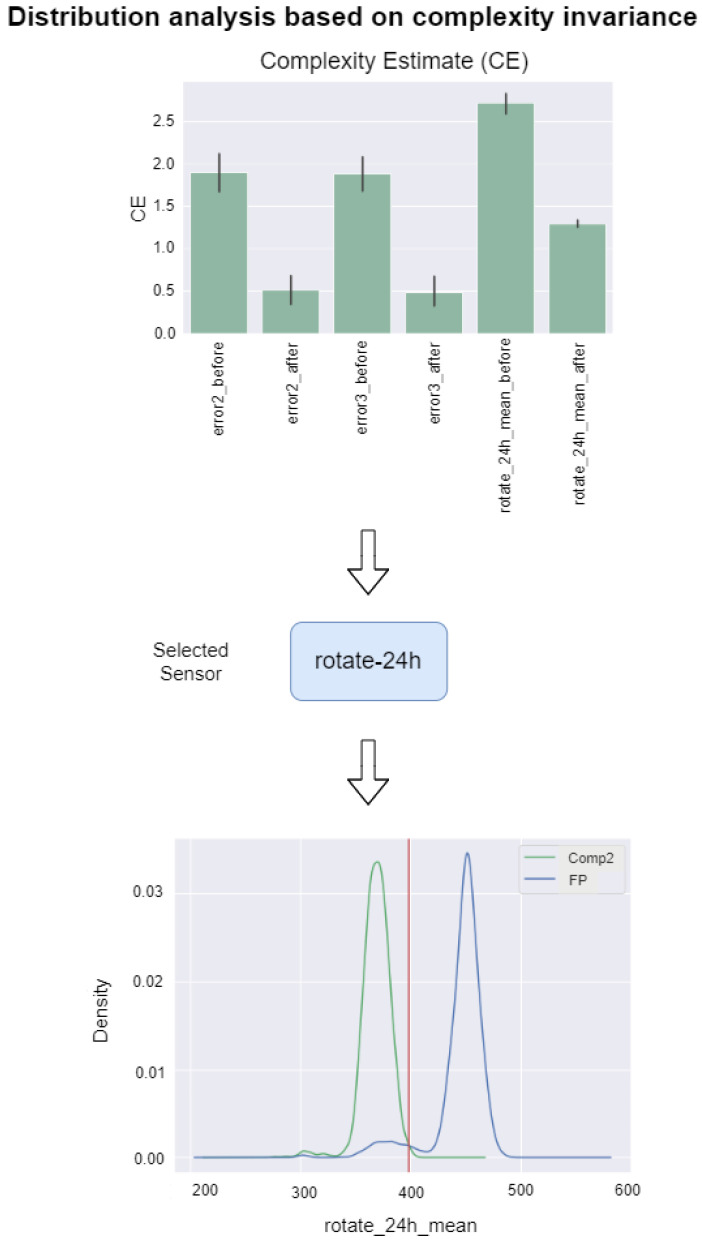
This example shows comp2 of Use Case 1. The distribution analysis is split into three consecutive steps of analysis of the feature ratio before and after the known maintenance events based on ce_ratio and ci_ratio, selection of the most informative feature, and definition of distribution threshold.

Figure 3 depicts the process of distribution threshold determination for comp2 in Use Case 1 by selecting the most informative feature “rotate-24h” and the distribution threshold of 405 to distinguish between FP events and real events. The bar chart on the top depicts the CE(X) metric for the features in green and Confidence Interval (CI) as the small back bars on top. The CI is estimated by bootstrapping [38]. The results are visualized using a bar chart with a confidence interval. The results are shown for each feature before and after a known maintenance event. The signal “rotate-24h” representing the averaged value of the “rotate” signal in 24 h is ranked as the most relevant signal based on the highest value of rank=ce_ratio+ci_ratio. The selection of “rotate-24h” is due to the fact at both its CE(X) and CI decrease, indicating that the signal is fluctuating less after the maintenance action. Hence, the effects of the performed maintenance action manifest themselves in this signal, and thus this signal ranks as the most relevant signal for distribution threshold analysis. Finally, the intersection point between the distributions of FP and real maintenance events is defined. Figure 3 shows the approach for comp2, but the approach is applied for every component in both use cases.

## 5. Experimental Results of Use Case 1

The evaluation for Use Case 1 compares performance indicators of four setups showing the benefits that post-filtering in MEDEP poses over unfiltered PELT. The baseline for the comparison conducted here are event detection results using only PELT and no post-filtering. Then, there are the two intermediate variants of using PELT and only the distribution threshold as post-filtering or using PELT and only the mean ratio as post-filtering. The final variant is PELT with the consecutive post-filtering by the mean ratio and the distribution threshold, creating the full pipeline of MEDEP. In addition to PELT, LOF as a promising approach applied for anomaly detection in manufacturing showed superior results [31]; therefore, it is included in the evaluation as a point of reference. The evaluation scores include sensitivity, FP rate, and accuracy. The sensitivity score indicates the true positive detection, the FP rate highlights the remaining share of FP in the results, and the accuracy is a combined measure showing the number of true detected events divided by the number of all detected events. We present the detection results for each component separately to outline the results clearly.

The results of the four evaluations of each component are depicted in Table 4 and show that MEDEP outperforms the cases with no or only one post-filtering. Therefore, MEDEP improves the accuracy and consequently reduces the FP rate without major influences on the sensitivity rate. These results are promising for applications where a low FP rate is required in order to gain trust and acceptance when integrating this approach in manufacturing environments. Therefore, the extended approach outperforms the PELT by keeping its original high sensitivity while reducing the FP rate at the same time.

MEDEP outperforms PELT and LOF in terms of FP rate when aiming for maintenance events detection. PELT and LOF introduce a high number of FPs since they catche any type of anomalies present in sensor data. A post-filtering approach similar to MEDEP could help to reduce the number of FPs also of LOF. In general, MEDEP clearly introduces fewer FP events, and this is an important aspect when aiming to increase the acceptance rate of ML approaches for maintenance event detection in real-world use cases.

## 6. Experimental Results of Use Case 2

This use case centers around welding and condition monitoring data collected in an industrial welding process. In this use case, we evaluate the MEDEP on the data from a single machine. The results of MEDEP are promising in terms of sensitivity.

Table 5 shows the results for a single machine and showing a drop in FP rate of 20% when post-filtering is applied. Again, the four setups are presented with no, partial, and full post-filtering. The results show that the proposed framework can detect already documented events with a high sensitivity rate. Overall, the results are promising in the context of maintenance detection, but only a small number of the maintenance events of component 1 were available for the evaluation. Therefore, we have to take this fact into consideration while interpreting the results. In general, the results are promising, and due to the good results in Use Case 1, this approach is sound for the detection of maintenance events. Still, the FP reduction is much smaller than in Use Case 1. The discussion of these results with domain experts generated the hypothesis that the undocumented maintenance events, such as minor cleanings and adjustments, show up here as FP events.

## 7. Discussion of Results and Outlook

Data-driven ML approaches can improve the detection of maintenance events or help with the labeling of data for PdM modeling [7,8,29]. Previous studies have focused mainly on improving labeling in the context of defect detection [9,10] and failure detection [11,12]. However, maintenance event detection has not been extensively explored so far. Moreover, the core focus of existing research was the detection of potential failures manifested in a signal rather than the retrospective detection of maintenance events. Additionally, none of the current approaches consider PELT in the context of maintenance events detection to help complete existing maintenance data-sets. This work designed an extended PELT approach providing fast and accurate maintenance event detection.

### 7.1. Theoretical Contributions

The high quality of the automatically detected maintenance events allows them to be used as inputs for other ML algorithms building PdM models. Our paper has two main contributions. Firstly, we demonstrated how the proposed framework called MEDEP addresses the challenge of having limited maintenance data, thus helping to automatically detect maintenance events. Secondly, we showed how the complexity estimate CE(X) extracts valuable knowledge concerning maintenance events, thus helping to identify and select relevant features for event detection.

Regarding the first contribution, the proposed MEDEP framework showed that maintenance events could be detected in sensor data. The high FP is a common challenge in the literature concerning event detection [15,16,17]. This challenge can hinder the application of event detection models in real-world use cases [17]. MEDEP shows high accuracy and low FP rate, giving it the foundations to be applied to maintenance event detection in a real-world use case. In general, our proposed solution reduces the average FP rate from 90% of a pure PELT approach to 10%, which is more applicable in real world scenarios. A low FP rate is crucial for the integration in real-world use cases where MEDEP increases the number of annotated maintenance events. Susto et al. [31] compared state-of-the-art anomaly detection approaches using different industrial use cases, where LOF showed superior results. However, our evaluation showed that MEDEP overcomes LOF in terms of FP rate. One aspect that contributed to these results is that MEDEP is specialized anomaly detection in maintenance events detection, i.e., interventions. LOF is a general anomaly detection approach and aims to catch any type of anomaly, thus leading to a higher FP rate. However, the foundation provided by MEDEP by introducing the post-filtering approach idea could be easily merged with any anomaly detection approach, e.g., LOF, to help reduce the FP rate. This can be considered as a promising direction for future research.

The second contribution is the application of the complexity estimate CE(X) to select the most informative features for the maintenance event detection. The contribution of this metric is twofold. Firstly, the metric is used for decision-making on multivariate feature selection. Secondly, this metric helped to select the most relevant feature in the post-filtering process. Employment of CE(X) to extract information concerning performed maintenance events is a novel application.

### 7.2. Limitations and Future Research Direction

MEDEP is applicable in cases where a lot of sensor data are collected, but only partly maintenance data are provided. However, MEDEP provides a high potential to be integrated as a supportive tool within a real-world use case. In this case, the MEDEP will detect potential candidate events from sensor data and the completeness of maintenance data will increase, thus paving the way for modeling PdM approaches [39]. This remains as an avenue for future work. Moreover, MEDEP is evaluated only on the detection of maintenance interventions. To generalize MEDEP further, a comparison and evaluation of the MEDEP against state-of-the-art anomaly detection approaches is required. Furthermore, the Multi-Component System (MCS) view of modeling interdependencies between components has been promoted as a promising approach to increase predictive results and decision-making performance in the context of PdM [1,40]. However, the main challenge highlighted in the literature is the lack of sufficient maintenance data to model the MCS view [1,9,40]. Especially, the deep component level required to build the MCS view increases the amount of required labeled data. MEDEP can help to tackle this challenge by increasing the completeness of maintenance event data. In this regard, we strongly believe that this will encourage researchers to further explore MEDEP.

MEDEP is based on PELT as an offline CPD approach focused on signal segmentation, promising a low computation time and high accuracy in the detection of change points. This approach showed superior results in similar case studies where the signal data are available. However, the integration of MEDEP in real-world use cases is planned as future work. In this case, other approaches intended primarily for real-time event detection, such as the online CDP approaches, can be extensively analyzed and benchmarked against MEDEP. MEDEP showed superior results in Use Case 1 in the ability to transfer the knowledge over the machines, i.e., training and testing in different machines. However, the transfer over machines in Use Case 2 is not evaluated as a result of considerably varying patterns of the same maintenance type over different machines. One promising approach to tackle this challenge is the adaptive normalization of the data for non-stationary heteroscedastic time series [41]. In the future, we plan to explore more in this direction.

## 8. Conclusions

In this research work, we exemplify how to identify maintenance events from sensor data. We proposed MEDEP as a novel maintenance event detection framework providing a high accuracy at a low FP rate. MEDEP is evaluated in two different industrial Use Cases, namely the Microsoft Azure Predictive Maintenance data-set and data from a real-world use case from the welding industry. Moreover, a metric based on a complexity estimate for time series is proposed for feature selection and distribution analysis in the context of maintenance event detection. MEDEP showed that it could reach a superior accuracy and low FP rate results, thus promising a high acceptance and application rate. In contrast to the benchmark ML anomaly detection approaches, MEDEP showed superior results with detection maintenance events, i.e., interventions.

In the future, we plan to investigate anomaly detection approaches such as online CPD aiming for real-time maintenance event detection. Furthermore, the completeness of data as an outcome of the application of MEDEP will be used to build PdM models. In particular, we aim to explore the MCS view and the interdependencies between components in the future. Finally, we aim to integrate and evaluate this approach within a real-world use case.

## Figures and Tables

**Table 1:** An example of telemetry data in Use Case 1 with information about time (“Datetime”), machine (“machineID”), and the sensors data consisting of voltage (“Volt”), rotation (“Rotate”), pressure (“Pressure”) and vibration (“Vibration”).

Datetime	MachineID	Volt	Rotate	Pressure	Vibration
01.01.2015 06:00	1	176.22	418.5	113.08	45.09
01.01.2015 07:00	1	162.88	402.75	95.46	43.41
01.01.2015 08:00	1	170.99	527.35	75.24	34.18

**Table 2:** Selected features for Use Case 1 and 2.

Use Case	Component	Features
1	Comp1	volt_24h_mean, error1
1	Comp2	rotate_24h_mean, error2, error3
1	Comp3	pressure_24h_mean, error4
1	Comp4	vibration_24h_mean, error5
2	Comp1	ErrorCount, Kurtosis, Mean, Variance, STD

**Table 3:** Trained hyper-parameters.

Use Case	Component	Parameter	Value	Min	Max
1	Comp1	penalty	50	10	1000
		mean ratio	1.01	1.001	2
		dist threshold volt_24h	183	-	-
		window_size	12	6	48
1	Comp2	penalty	100	10	1000
		mean ratio	2.0	1.001	2
		dist threshold rotate_24h	405	-	-
		window_size	12	6	48
1	Comp3	penalty	45	10	1000
		mean ratio	1.1	1.001	2
		dist threshold pressure_24h	114.75	-	-
		window_size	12	6	48
1	Comp4	penalty	50	10	1000
		mean ratio	1.001	1.001	2
		dist threshold vibration_24h	46.99	-	-
		window_size	12	6	48
2	Comp1	penalty	100	10	1000
		mean ratio	1.5	1.001	2
		dist threshold variance	0.109	-	-
		window_size	50	5	150

**Table 4:** Maintenance event detection results in Use Case 1. The best results for each metric are highlighted in bold.

Component	Algorithm	Sensitivity	FP Rate	Accuracy	Distribution Threshold	Mean Ratio
Comp1	MEDEP	0.975	0.948	0.051	False	False
	MEDEP	0.975	0.768	0.231	True	False
	MEDEP	0.878	0.700	0.300	False	True
	**MEDEP**	**0.878**	**0.052**	**0.947**	True	True
	LOF	0.531	0.545	0.469	-	-
Comp2	MEDEP	0.943	0.936	0.063	False	False
	MEDEP	0.943	0.734	0.265	True	False
	MEDEP	0.943	0.572	0.472	False	True
	**MEDEP**	**0.943**	**0.122**	**0.877**	True	True
	LOF	0.467	0.527	0.473	-	-
Comp3	MEDEP	0.900	0.963	0.037	False	False
	MEDEP	0.900	0.858	0.142	True	False
	MEDEP	0.850	0.767	0.232	False	True
	**MEDEP**	**0.850**	**0.105**	**0.895**	True	True
	LOF	0.522	0.544	0.456	-	-
Comp4	MEDEP	1.000	0.908	0.092	False	False
	MEDEP	1.000	0.593	0.407	True	False
	MEDEP	0.945	0.313	0.687	False	True
	**MEDEP**	**0.945**	**0.054**	**0.946**	True	True
	LOF	0.407	0.461	0.539	-	-

**Table 5:** MEDEP maintenance event detection results for one component of a single industrial welding machine.

Component	Algorithm	Sensitivity	FP	Accuracy	Distribution Threshold	Mean Ratio
Comp1	MEDEP	0.750	0.900	0.100	False	False
	MEDEP	0.750	0.880	0.012	True	False
	MEDEP	0.750	0.750	0.250	False	True
	MEDEP	0.750	0.700	0.300	True	True
	LOF	0.500	0.980	0.010	-	-

## Data Availability

Due to the confidentiality of the data collected directly from a real use case, it is not possible to share the raw data. However, the data from Use Case 1 are public data and accessible in [33].
